# Transcriptomics analysis highlights potential ways in human pathogenesis in *Leishmania braziliensis* infected with the viral endosymbiont LRV1

**DOI:** 10.1371/journal.pntd.0012126

**Published:** 2024-05-14

**Authors:** Kátia Paula Felipin, Mauro Valentino Paloschi, Milena Daniela Souza Silva, Yoda Janaina Ikenohuchi, Hallison Mota Santana, Sulamita da Silva Setúbal, Cristina Matiele Alves Rego, Jéssica Amaral Lopes, Charles Nunes Boeno, Suzanne Nery Serrath, Enmanuella Helga Ratier Terceiro De Medeiros, Iasmin Ferreira Pimentel, Antonio Edson Rocha Oliveira, Elisa Cupolillo, Lilian Motta Cantanhêde, Ricardo de Godoi Matos Ferreira, Juliana Pavan Zuliani

**Affiliations:** 1 Laboratório de Epidemiologia Genética, Fundação Oswaldo Cruz, FIOCRUZ Rondônia, Porto Velho, Brazil; 2 Laboratório de Imunologia Celular Aplicada à Saúde, Fundação Oswaldo Cruz, FIOCRUZ Rondônia, Porto Velho, Brazil; 3 Núcleo de Biologia Experimental, Universidade de Fortaleza, UNIFOR, Fortaleza, CE, Brazil; 4 Laboratório de Pesquisa em Leishmanioses, Instituto Oswaldo Cruz, Fundação Oswaldo Cruz, FIOCRUZ, Rio de Janeiro, Brazil; 5 Instituto Nacional de Epidemiologia da Amazônia Ocidental, EpiAmO, Porto Velho, Brazil; 6 Departamento de Medicina, Universidade Federal de Rondônia, UNIR, Porto Velho, Brazil; Universidade Federal de Minas Gerais, BRAZIL

## Abstract

The parasite *Leishmania* (*Viannia*) *braziliensis* is widely distributed in Brazil and is one of the main species associated with human cases of different forms of tegumentary leishmaniasis (TL) such as cutaneous leishmaniasis (CL) and mucosal leishmaniasis (ML). The mechanisms underlying the pathogenesis of TL are still not fully understood, but it is known that factors related to the host and the parasite act in a synergistic and relevant way to direct the response to the infection. In the host, macrophages have a central connection with the parasite and play a fundamental role in the defense of the organism due to their ability to destroy intracellular parasites and present antigens. In the parasite, some intrinsic factors related to the species or even the strain analyzed are fundamental for the outcome of the disease. One of them is the presence of *Leishmania* RNA Virus 1 (LRV1), an endosymbiont virus that parasitizes some species of *Leishmania* that triggers a cascade of signals leading to a more severe TL phenotype, such as ML. One of the strategies for understanding factors associated with the immune response generated after *Leishmania*/host interaction is through the analysis of molecular patterns after infection. Thus, the gene expression profile in human monocyte-derived macrophages obtained from healthy donors infected *in vitro* with *L*. *braziliensis* positive (LbLRV1+) and negative (LbLRV1-) for LRV1 was evaluated. For this, the microarray assay was used and 162 differentially expressed genes were identified in the comparison LbLRV1+ vs. LbLRV1-, 126 upregulated genes for the type I and II interferons (IFN) signaling pathway, oligoadenylate synthase OAS/RNAse L, non-genomic actions of vitamin D3 and RIG-I type receptors, and 36 down-regulated. The top 10 downregulated genes along with the top 10 upregulated genes were considered for analysis. Type I interferon (IFNI)- and OAS-related pathways results were validated by RT-qPCR and Th1/Th2/Th17 cytokines were analyzed by Cytometric Bead Array (CBA) and enzyme-linked immunosorbent assay (ELISA). The microarray results validated by RT-qPCR showed differential expression of genes related to IFNI-mediated pathways with overexpression of different genes in cells infected with LbLRV1+ compared to LbLRV1- and to the control. No significant differences were found in cytokine levels between LbLRV1+ vs. LbLRV1- and control. The data suggest the activation of gene signaling pathways associated with the presence of LRV1 has not yet been reported so far. This study demonstrates, for the first time, the activation of the OAS/RNase L signaling pathway and the non-genomic actions of vitamin D3 when comparing infections with LbLRV1+ versus LbLRV1- and the control. This finding emphasizes the role of LRV1 in directing the host’s immune response after infection, underlining the importance of identifying LRV1 in patients with TL to assess disease progression.

## Author summary

*Leishmania* (*Viannia*) *braziliensis* is a parasite prevalent in Brazil and associated with tegumentary leishmaniasis (TL), including cutaneous (CL) and mucosal (ML) forms. The mechanisms of pathogenesis of TL are not fully understood, including some factors related to the host and parasite interaction in response to infection, and especially about *Leishmania* RNA Virus 1 (LRV1), an endosymbiont virus parasitizing *Leishmania* species, particularly triggers ML. Molecular approaches are usually applied to compare situations and to understand these interactions. Here, microarray analysis identified 162 differentially expressed genes in LbLRV1+ vs. LbLRV1- infection, with 126 upregulated genes related to IFN signaling, OAS/RNAse L, vitamin D3, and RIG-I type receptors. Additionally, 36 down-regulated genes were observed. Then, two validation assays were performed to confirm these results (RT-qPCR and Cytometric Bead Array). The main results comprise the differential gene expression in cells infected with LbLRV1+ compared to LbLRV1- and control, with overexpression of various genes in LbLRV1+ cells. Cytokine levels showed no significant differences between LbLRV1+ and LbLRV1-. This study highlighted the activation of the OAS/RNase L signaling pathway and the non-genomic actions of vitamin D3 in LbLRV1+ infection compared to LbLRV1- and control. This research contributes to our understanding of the immune response and molecular pathways involved in *Leishmania* infections, particularly in the presence of LRV1.

## 1. Introduction

Leishmaniases are a group of diseases present in 98 countries and are among the ten main neglected tropical diseases in the world. Annually, approximately 1 million new cases occur, about 20,000 to 30,000 deaths and 350 million people are at risk of infection [1,2].

In Brazil, the main species that cause tegumentary leishmaniasis (TL) are *Leishmania* (*Viannia*) *braziliensis* and *L*. (*V*.) *guyanensis* [3]. In the Amazon region, in addition to these species, five others have been attributed to cases of TL in humans: *L*. (*V*.) *lainsoni*, *L*. (*V*.) *naiffi*, *L*. (*V*.) *lindenbergi*, *L*. (*V*.) *shawi* and *L*. (*Leishmania*) *amazonensis* [4–9]. In the Americas, *Leishmania* (*Viannia*) *braziliensis* is the most widely distributed species associated with cutaneous, mucosal, and *disseminated* forms of the disease. In Brazil, in addition to *L*. *braziliensis*, seven other *Leishmania* species are associated with human cases of cutaneous or visceral leishmaniasis, all these present in the Brazilian Amazonia region [10].

The initial contact of *Leishmania* with components of the immune system during the initial stages of the infection plays a fundamental role, which can lead to self-control of the disease or its development [11]. Literature has shown that macrophages play an essential role in infection by *L*. *braziliensis*, since depending on the chemokines and inflammatory cytokines secreted, they can direct both a protective response to the host and the development of the pathology [12,13]. Analyzes carried out from infections of PBMCs with *L*. *braziliensis* showed that monocytes play an important role in the development of the disease, as the frequency of monocytes in individuals infected by this strain increased. In addition, it was seen that monocytes express CCR2 (CC chemokine receptor type 2) and produce TNF (Tumor Necrosis Factor) which can promote the migration of leukocytes to the injured site, intensifying the inflammatory response contributing to the development of the ulcer [14].

From the *in vitro* exposure of human peripheral blood mononuclear cells (PBMCs) to *L*. *braziliensis*, two patterns of stable IFN-γ (Interferon-gamma) production can be developed, high-level (high responder—HR) or low-level (low responder—LR), presenting different genetic signatures. CXCL10, IFI27, IL6, and LTA genes were positively regulated in both HR and LR individuals. In addition to these, it was identified that the expression of the genes CCL7, IL8, IFI44L, and IL1β are linked to the HR pattern that presents correlations with the IL17 and TREM1 signaling pathways. On the other hand, the IL9, IFI44, IFIT1, and IL2RA genes were expressed exclusively in the LR condition and were associated with pathways related to pattern recognition and interferon signaling receptors [15].

It is known that *L*. *braziliensis* strains can harbor the *Leishmania RNA Virus* 1 (LRV1), which contributes to the modulation of the human innate immune response, favoring the parasite survival and worsening of the disease. The mechanisms by which *Leishmania* with LRV1 aggravates TL are still not fully understood. The LRV1 recognition via TLR3 culminates in the increase of inflammatory cytokines, TNF-α and IL-12, and the autophagy, that degrades NLRP3 and ASC, NLRP3 inflammasome proteins [16]. Furthermore, the virus promotes the production of TLR3-mediated inflammasome-independent inflammatory cytokines, which may contribute to the development of mucocutaneous lesions in patients. It is important to mention that the activation of the NLRP3 inflammasome limits the replication of *Leishmania* in macrophages, which leads to the development of an evasion strategy [17].

Studies have demonstrated that the presence of LRV1 in strains of *L*. *braziliensis* and *L*. (*Viannia*) *guyanensis* significantly contributes to the exacerbation of the immune response and an increase in parasitic load, both in experimentally infected animals and in humans. However, it is still not well-established what genetic mechanisms are triggered by LRV1 to modulate the immune response during *Leishmania* infection [16,18,19].

In recent years, technological advances have contributed to the development of high-throughput methodologies and robust bioinformatics tools that allow the identification of differentially expressed genes and the prediction of associated regulatory pathways and networks. The dataset can be made available in public repositories and reused [20]. In this context, transcriptomic analysis has the potential to contribute to the identification of therapeutic targets, pathogenic mediators, and new biological insights, which leads to the generation of new hypotheses about the pathogenic mechanisms of complex diseases.

To this end, this study aimed to evaluate the expression of differentially expressed genes in human macrophages-derived monocytes infected *in vitro* with an *L*. *braziliensis* bearing LRV1 (LbLRV1+) strain concerning the expression of macrophages-derived monocytes infected with an LbLRV1- strain and to correlate the findings to biological processes through pathway enrichment.

## 2. Material and methods

### 2.1. Study population and ethics statement

The Center for Research in Tropical Medicine Ethics Committee approved this research (CAAE 13655519.6.0000.0011). Individuals who agreed to participate were informed about the purpose of the study and the scientific relevance of the work verbally and in writing, and their participation was formalized by signing the Informed Consent Form. Male donors between 18 and 40 years old, originating from non-endemic areas for *Leishmania*, were selected and those who reported recent infections or drug treatment to preserve cellular analysis of peripheral blood were excluded.

### 2.2. Parasites

The *L*. *braziliensis* strains used to carry out the experiments (LbLRV1+ MHOM/BR/2015/RO475, IOCL3621 and LbLRV1- MHOM/BR/2014/RO314, IOCL3626) were isolated from patients attended at Reference Center in Tropical Diseases in Rondonia (CEMETRON) with a diagnosis of leishmaniasis, and the species characterization was conducted and confirmed at the Fiocruz *Leishmania* Collection (CLIOC) by isoenzyme electrophoresis (MLEE). It is important to highlight that *Leishmania* isolates present genotypic variation, that is, they are not isogenic. *Leishmania* promastigotes were cultivated in Schneider´s medium (Gibco, Paisley, Scotland, UK) supplemented with 20% fetal bovine serum (Gibco, United States) and 2% filtered human urine, incubated at 25°C in a Biochemical Oxygen Demand for 5 days when the parasites reached the stationary phase. For LRV1 characterization, 1x10^7^ parasites of each culture were centrifuged at 4000 x *g* for 10 min for RNA extraction. The RNeasy Mini kit (Qiagen) was used. The cDNA synthesis was performed with iScript Reverse Transcription Supermix kit (BioRad) from 1 μg of RNA, following the manufacturer’s protocol. To detect and quantify LRV1, the primers LRV1 76 F 5’- GACTGATTGGACGGAGGGCA -3’ and LRV1 76 R 5’-TGCTGTGGAACGTGAGGAACT-3’ that amplify ORF1 LRV1 genome were used. Ten μL of iTaq Universal SYBR Green Supermix (Bio-Rad Laboratories, USA), 6.8 μL of water, 0.6 μL of primer F (0.3 μM), 0.6 μL of primer R (0.3 μM), and 2 μL of cDNA were used per reaction. The cycling parameters for the reactions were 50°C for 2 min, 95°C for 10 min, and then 40 cycles of 95°C for 15 s and 64°C for 1 min. The dissociation curve was performed at 62°C for 1 min.

### 2.3. PBMCs isolation and culture

The peripheral blood mononuclear cells (PBMCs) were isolated by Ficoll-Paque PLUS (GE Healthcare Bio-Sciences AB, Uppsala, Sweden) through density gradient centrifugation. The same amount of Ficoll-Paque PLUS (GE Healthcare Bio-Sciences AB, Uppsala, Sweden) and whole blood diluted 1:1 in 1X phosphate-buffered saline (PBS) was placed in 50 mL sterile tubes and processed according to the manufacturer’s instructions. PBMCs were dispensed into 24-well plates at a concentration of 5x10^6^ cells in 1 mL of RPMI culture medium supplemented with gentamicin (40 mg/mL), L-glutamine (2 mM/L), and 10% donor’s serum per well.

### 2.4. PBMCs immunophenotyping

To assess the viability of PBMCs, 1x10^6^ cells/per well with a final volume of 100 μL of RPMI-supplemented medium with gentamicin (40 mg/mL), L-glutamine (2 mM/L) and 10% donor serum were labeled with 0.1 μL of propidium iodide (PI) and 0.2 μL of thiazole orange (TO) (BD Cell Viability Kit) followed by vortexing and incubation for 5 min at room temperature. As a negative control, PBMC cultivated only with RPMI was used, and cells killed by ethanol were used as a positive control. In parallel, CD14 was labeled with PE-conjugated anti-CD14 (Phycoerethrin) for 30 min on ice and protected from light. 60,000 events were acquired on the FACScan flow cytometer (Becton Dickinson Immunocytometry Systems, San Jose, CA, USA) using the CELLQuest software followed by analysis on FlowJo vX.0.7 (Becton Dickinson, USA). After acquisition, cells were selected by forward scatter (FSC) and side scatter (SSC) following the label of interest. Separate staining was performed to analyze the monocyte population (CD14) and cell viability (PI/TO). The results were expressed as percentages.

### 2.5. Cells infection

PBMCs in supplemented RPMI medium were dispensed in 24-well microplates on sterile coverslips (13mm) and kept for 7 days in a controlled incubator at 37°C and 5% CO_2_ [21]. After 2 h and 24 h, the PBMCs were washed three times with RPMI and re-incubated with 1 mL of supplemented RPMI medium. In the culture medium (RPMI supplemented with 10% inactivated autologous serum) [22] no other exogenous agent was added and the differentiation of monocytes into macrophages was performed by the plastic adherence method, which selects the population of monocytes through cell adherence to the artificial substrate [23]. Adhered cells were washed three times with RPMI and subjected to the infection process. For the infection of human monocyte-derived macrophages with *L*. *braziliensis* promastigotes, the MOI 10:1 (parasites: macrophage) was used and the plates were incubated at 34°C and 5% CO_2_ for 24 h. Cells were then washed three times with RPMI to remove non-internalized parasites and then re-incubated for 72 h under the same conditions. A plate containing only the PBMC culture was maintained under the same conditions that were used for the *in vitro* infection to allow comparison between infected and uninfected plates. After the incubation and infection period, the coverslips placed in the 24-well plates were removed and stained with Instant–Prov (New Prov) according to the manufacturer’s instructions and fixed with Entellan (MERCK) on slides for microscopy. 100 cells were observed per slide, counted by optical microscopy in a 100X objective. To determine the phagocytic index, the percentage of infected macrophages in 100 total cells visualized was considered, multiplying the average number of amastigotes by macrophages [24].

### 2.6. RNA isolation and microarray hybridization

Seventy-two h post-infection (PI), human monocyte-derived macrophages were washed three times with sterile 1X PBS (pH 7.4) [25,26]. Total RNA was separated using Trizol (Life Technologies, California, EUA) followed by extraction with the *RNeasy Mini kit* (Qiagen, Hilden, Germany) to perform the microarray reaction and RT-qPCR, according to the manufacturer’s instructions. RNA concentration and purity were evaluated using the NanoDrop 2000 spectrophotometer (Thermo Fisher Scientific, Waltham, Massachusetts). The RNA was processed using the GeneChip WT PLUS Reagent Kit (Thermo Fisher Scientific) for purification, reverse transcription, fragmentation, and labeling of the samples, followed by application in a Clariom S Assay Human (Thermo Fisher Scientific, EUA), according to the manufacturer’s recommendations.

### 2.7. Microarray data analyses

The raw data has been submitted to the NCBI Gene Expression Omnibus (GEO) and is available under accession number GSE247487. The CEL files from the Affymetrix platform (*GPL23159*) related to 27189 probes were read and normalized by the oligo package [27] using the R version 4.1.2 programming language. The quality of 15 samples (3 groups and 5 donors) was analyzed using the arrayQualityMetrics package [28] to identify possible outliers and remove them from downstream analyses. Probes without annotation or related to more than one gene were removed. For genes that presented more than one probe, the most expressed probe was selected resulting in a total of 18478 probes/genes analyzed. The differential expression analysis pipeline followed the instructions of the limma package for single-channel microarray studies [29], performing linear modeling accompanied by empirical Bayesian statistics. The false discovery rate method was used to adjust p values for multiple tests. The experimental design was created using groups for comparisons and considering donors as a batch effect. Three comparisons were performed to identify the different biological meanings related to the samples: 1) cells infected with *L*. *braziliensis* LRV1-negative versus control (LbLRV1-/control) to identify genes related to infection without the influence of LRV; 2) cells infected with *L*. *braziliensis* LRV1 positive versus control (LbLRV1+/control) to identify genes related to the infection affected by LRV, and 3) cells infected with *L*. *braziliensis* LRV1 positive versus *L*. *braziliensis* LRV1 negative (LbLRV1+/ LbLRV1 -) to focus on genes related to the presence of LRV without the bias of *leishmania* infection. The average of the replicates of each group was compared to obtain the fold change in expression between the comparisons performed. Differentially expressed genes were identified based on adjusted p-value <0.05 and defined as upregulated (≥ 0.5) and downregulated (≤ -0.5) based on log2 fold change. Common and unique genes related to each comparison are provided in S1 Table. The top 10 genes were selected based on the list of differentially expressed genes provided in S1 Table and which presented the highest fold change values related to each comparison. To investigate the biological significance of upregulated genes, pathway enrichment was performed in EnrichR. The pathways were considered enriched when presented adjusted p-value < 0.05.

### 2.8. RT-qPCR

To confirm the expression of ISG15, IFIT1, IFIT2, IFIT3, IFITM3, IFI6, OAS1, OAS2, OAS3, and OASL genes, RT-qPCR was performed. For this assay, BioRad iScript Reverse Transcription Supermix kits for complementary DNA synthesis, and iTaq Universal SYBR Green Supermix (Bio-Rad Laboratories, EUA) were used on Rotor-Gene Q 5 Plex HRM (Qiagen N.V., EUA), with pre-designed primers for expression gene for mRNA analysis (DNA Express Biotechnology) (S3 Table). A 10 ng of RNA in all experimental conditions was used. The cells used as reference were macrophages derived from human monocytes without *Leishmania* strains (control) and glyceraldehyde 3-phosphate dehydrogenase (GAPDH) as an endogenous reference gene was used and expression values were determined by the 2ΔΔCt method. qPCR efficiency values for each of the evaluated genes are equivalent to the qPCR efficiency performed for GAPDH, making the data reliable for estimating gene expression [26,30].

### 2.9. ELISA cytokines measurements

At 24, 48, 72, 96, and 120 h PI, supernatant of all conditions was collected and stored at -20°C for cytokine quantification. Human IFN-α All Subtype (R&D Systems, EUA), Human IFN-β (R&D Systems, EUA), and Human CCL18/PARC (R&D Systems, EUA) were used according to the manufacturer’s instructions. Results for each cytokine were expressed in pg/mL.

### 2.10. CBA cytokines measurements

At 24, 48, 72, 96, and 120 h PI, supernatant of all conditions was collected and stored at -20°C for Interleukin-2 (IL-2), Interleukin-4 (IL-4), Interleukin-6 (IL-6), Interleukin-10 (IL-10), Tumor Necrosis Factor (TNF), Interferon-gamma (IFN-γ) and interleukin-17A (IL-17A) protein levels measured in a single sample using the CBA Human Th1/Th2/Th17 Cytokine Kit (BD Biosciences, San Diego, CA) according to the manufacturer’s instructions. Results were expressed in MFI (Mean Fluorescence Intensity).

### 2.11. LDH quantification

At 24, 48, 72, 96, and 120 h PI, supernatant of all conditions was collected and stored at -20°C for LDH (Lactate Dehydrogenase) quantification using the LDH Liquiform kit according to manufacturer instructions. Initial absorbance was registered after 1 min and the second was registered after 2 min from the first one. Absorbances were conducted in BioTek Synergy HT Multi-Detection (Winooski, VT) with 340 nm. Results were expressed in U/L.

### 2.12. Statistical analysis

The graphs were plotted using GraphPad Prism Ver 7.04 (GraphPad Software Incorporated). The results were expressed as mean ± standard error and statistically analyzed by Student’s t-test or analysis of variance (ANOVA) followed by Dunnett’s or Tukey’s post-test. Values of p<0.05 were considered statistically significant.

## 3. Results

### 3.1. *In vitro* infection of human monocyte-derived macrophages

**Fig 1:**
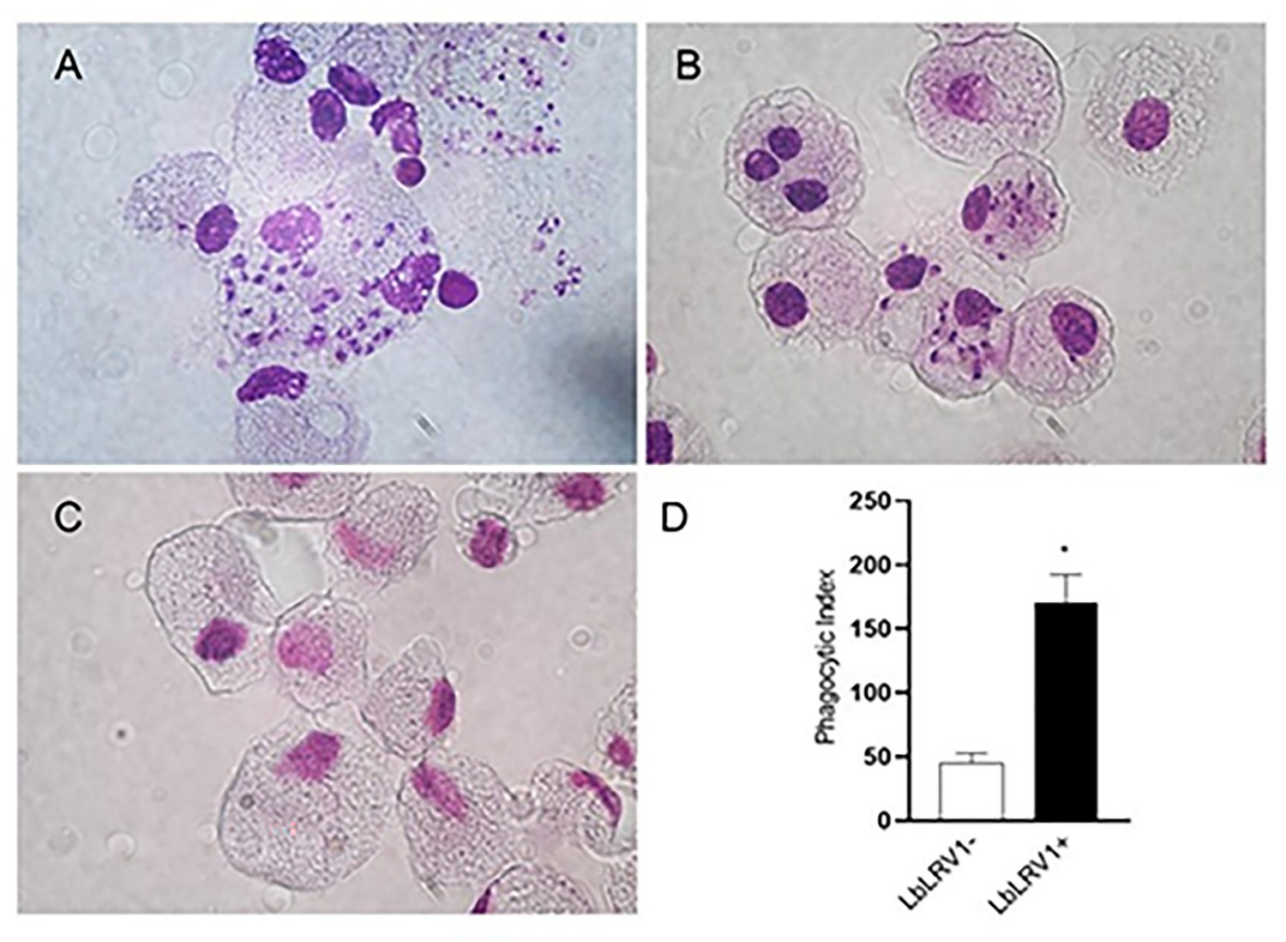
*In vitro* macrophage infection with *L* (*V*.) *braziliensis* with and without the viral endosymbiont (LRV). (**A**) human monocyte-derived macrophages infected with LbLRV1+; (**B**) human monocyte-derived macrophages infected with LbLRV1-; (**C**) control and (**D**) phagocytic index. Values represent the mean and standard error of 3 donors. *P<0.05, compared to the presence of the LRV1 viral endosymbiont (data presented with *T*-test).

To carry out infection assays with the LbLRV1+ and LbLRV1- strains, blood samples were collected from fourteen healthy donors to obtain PBMCs. The blood was diluted 1/1 in 1X PBS and a high percentage of PBMCs (90.8%, S1A Fig) was obtained from the isolation procedure (S1 Fig). In S1D Fig, it is possible to observe that 24.6% of CD14+ cells (monocytes, FL2) were viable (99.1%, S1B Fig). In Fig 1, it is possible to observe the human monocyte-derived macrophages infected with LbLRV1+ (Fig 1A) presented a high number of amastigotes inside the cells. The cells infected with LbLRV1- (Fig 1B) presented few amastigotes in the cytoplasm and Fig 1C shows the control human monocyte-derived macrophages without *Leishmania* strains. Fig 1D presented the phagocytic index showing that when human monocytes-derived macrophages were infected with amastigotes of LbLRV1+ this index increased compared to human monocytes-derived macrophages infected with LbLRV1-.

### 3.2. Transcriptome analysis of human monocyte-derived macrophages infected with LbLRV1+ or LbLRV1-

The data obtained from 15 chips from 5 donors (D1, D2, D3, D4, and D5) were initially evaluated, and of these, nine chips presented satisfactory quality metrics. The transcriptome of human monocyte-derived macrophages from 3 donors (D3, D4, and D5) infected with LbLRV1+, LbLRV1-, and control were analyzed. Despite the limitation of the study due to the sample size of monocyte donors and *Leishmania* isolates, the presented data remains highly relevant to the scientific community. Gene expression analysis by microarray allows for a comprehensive study of gene behavior, comparing the two conditions presented here: infection of human monocytes with LbLRV1+ or LbLRV1-, enabling the identification of expression patterns and affected biological pathways, providing valuable insights into the underlying biology.

S1 Table illustrates all differentially expressed genes analyzed by each comparison, as well as common and unique genes represented in the Venn diagram. As reducing the number of genes impacts the identification of enriched pathways, we decided to analyze pathways only for the gene sets related to each comparison. S2 Table contains results regarding pathway enrichment for each comparison.

**Fig 2:**
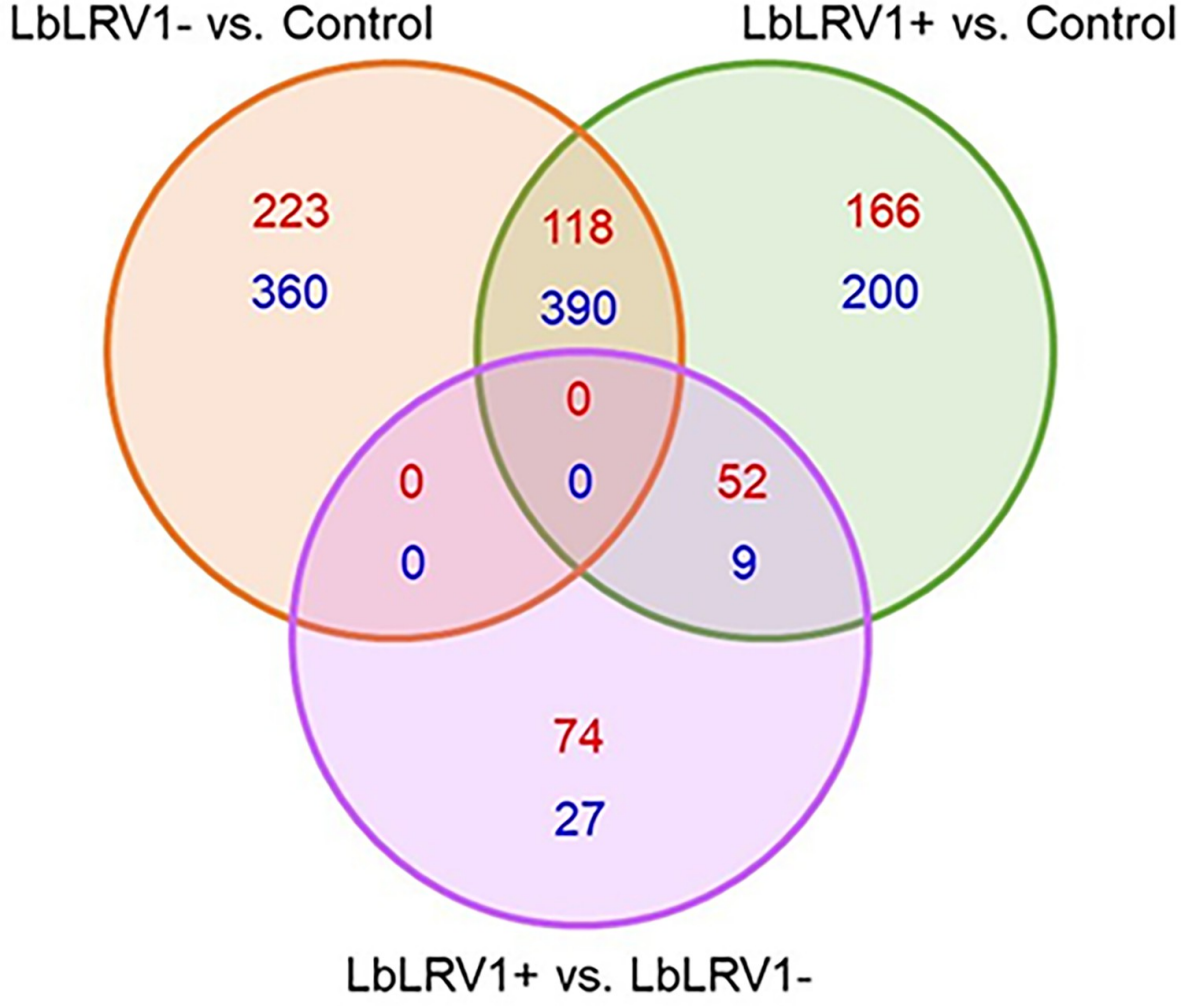
Venn diagram of the compared groups. Similarities between groups are represented in the overlapping portions of the circles, while differences are represented in the non-overlapping portions of the circles from LbLRV1- vs. control, LbLRV1+ vs. control and LbLRV1+ vs. LbLRV1- groups showing inside the circles the upregulated genes in red and downregulated genes in blue.

The Venn diagram (Fig 2) shows how many genes were differentially expressed in the three groups compared: LbLRV1- vs. control (223 upregulated and 360 downregulated), LbLRV1+ vs. control (166 upregulated and 200 downregulated) and LbLRV1+ vs. LbLRV1- (74 upregulated and 27 downregulated). The results were expressed in a heatmap and a volcano plot with upregulation (upregulation–red color) and negative regulation (downregulation–blue color) based on comparisons between conditions with and without human monocytes-derived macrophage stimulation.

**Fig 3:**
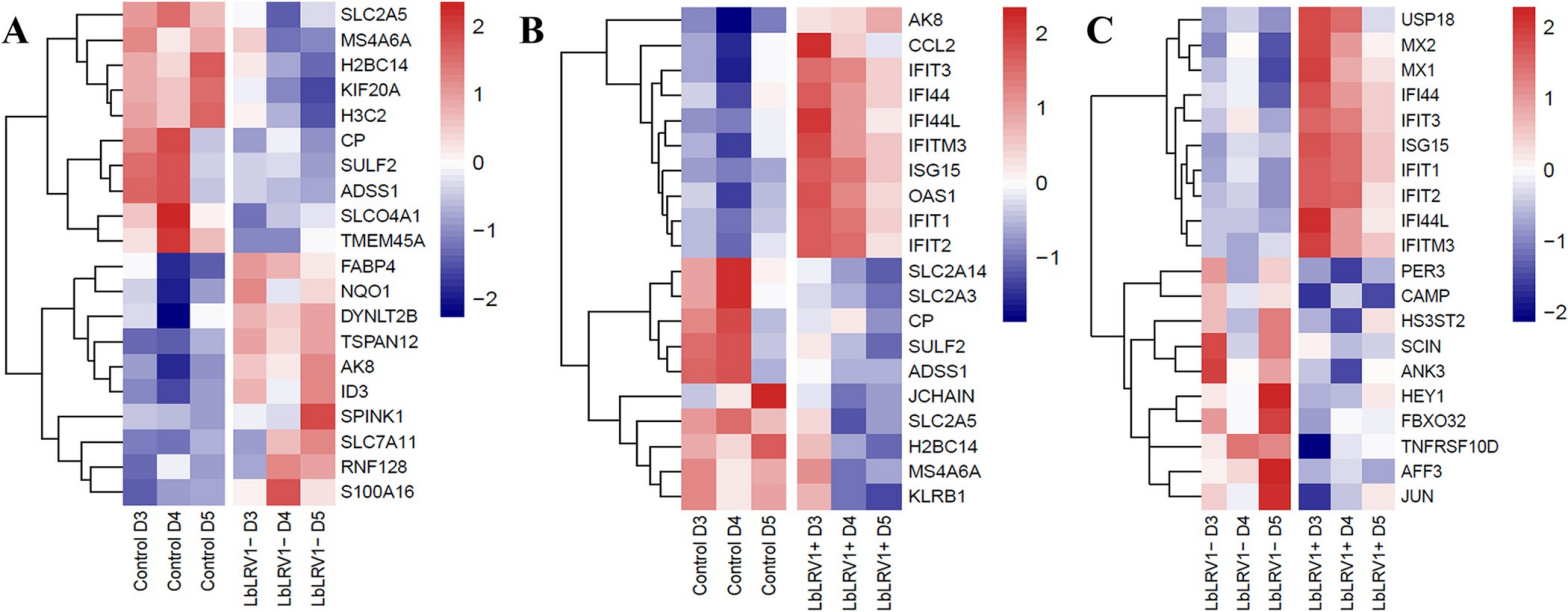
Heatmap of the top 10 up and downregulated genes. (**A**) Comparison between LbLRV1- vs control, (**B**) LbLRV1+ vs control, and (**C**) and LbLRV1+ vs. LbLRV1-. Genes with high expression are indicated in red and low expression are indicated in blue. Hierarchical clustering was performed using the Euclidean distance to obtain gene clusters.

**Fig 4:**
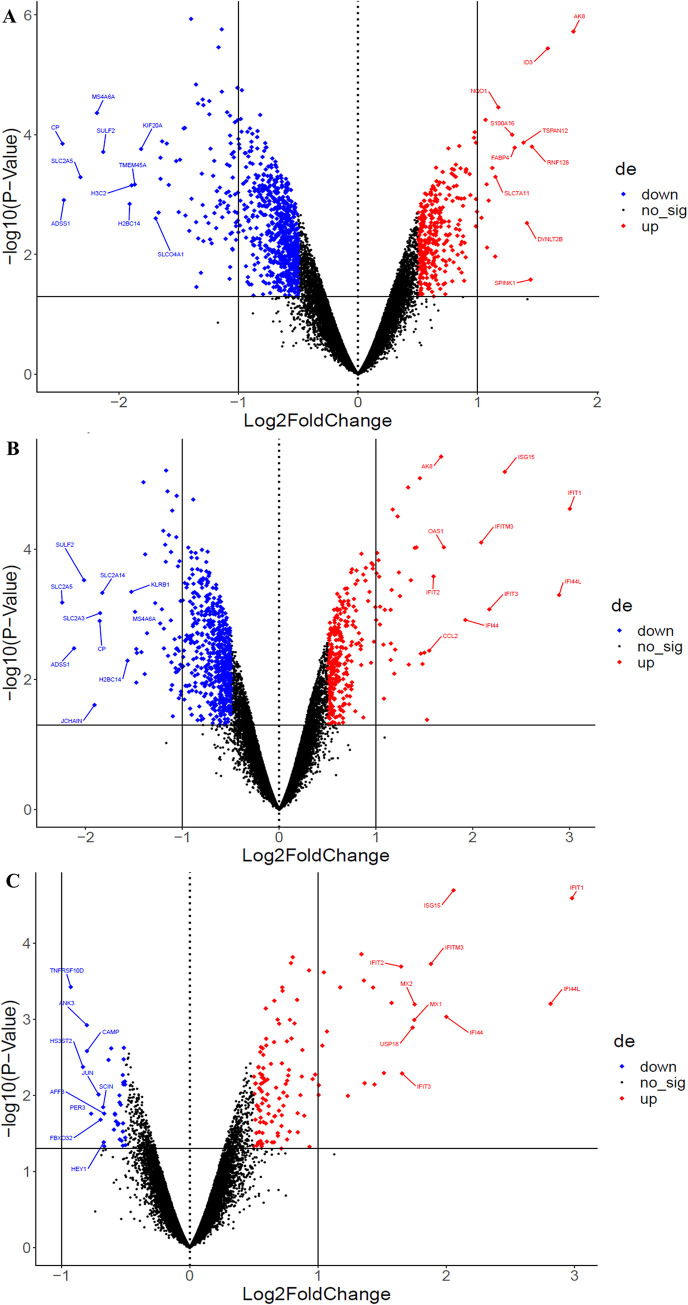
Volcano plots of the top 10 up and downregulated genes from LbLRV1- vs. LbLRV1- vs. control (**A**), LbLRV1+ vs. control (**B**), and LbLRV1+ vs. LbLRV1- (**C**). Upregulated genes are indicated in red and the downregulated ones in blue.

Comparing LbLRV1- vs. control identified 1091 differentially expressed genes, 341 upregulated and 750 downregulated. Comparing LbLRV1+ vs. control detected 935 differentially expressed genes, 336 upregulated and 599 downregulated. And, comparing LbLRV1+ vs. LbLRV1- demonstrated 162 differentially expressed genes, with 126 upregulated and 36 downregulated. From these genes, the top 10 upregulated and top 10 downregulated genes from each group were selected and compared as depicted in the heatmap in Fig 3 and the volcano plot in Fig 4.

Although each of these genes has different functions, there may be an indirect relationship between some of them since many biological processes are interdependent and regulated by different signaling pathways. Some of these genes may be involved in similar or related processes. For better organization, the data were tabulated with the groups of genes selected for the respective comparisons (LRV1-/control; LRV1+/control; LRV1+/LRV1-), grouped by functions, with their respective references, as shown in Tables 1–3.

**Table 1:** Comparing LbLRV1- vs. control, among the top 10 overexpressed genes and the top 10 under-expressed genes.

Genes	Mechanism	Reference
**Overexpressed**		
FABP4	Lipid metabolism	[31]
NQO1	Oxidative stress response	[32]
SLC7A11	Antioxidant response	[33]
SPINK1	Regulation of proteases	[34]
DYNLT2B	“Retrograde” cellular motor	[35]
TSPAN12	Co-receptor that amplifies frizzled4 ligand selectivity and signaling	[36]
AK8	Involved in nucleoside diphosphate Phosphorylation and nucleoside triphosphate biosynthetic process	[37]
ID3	Inhibitor of E protein transcription factors	[38]
RNF128	Inhibiting NF-κB activation	[39]
S100A16	Regulator of the inflammatory response	[40]
**Under-expressed**		
SLC2A	Related to different biological processes and diseases	[41]
MS4A6A	Related to different biological processes and diseases	[42]
CP	Related to different biological processes and diseases	[43]
SULF2	Related to different biological processes and diseases	[44]
SLCO4A1	Related to different biological processes and diseases	[45]
TMEM45A	Related to different biological processes and diseases	[46]
H2BC14	Nucleosome remodeling	[47]
H3C2	Chromatin structure	[48]
ADSS1	Purine nucleotide synthesis	[49]
KIF20A	Cell division	[50]

**Table 2:** Comparing LbLRV1+ vs. control, among the top 10 overexpressed genes and the top 10 under-expressed genes.

Genes	Mechanism	Reference
**Overexpressed**		
IFIT3	Are related to the viral immune response and are addressed in the discussion	
IFITM3		
ISG15		
OAS1		
IFIT1		
IFIT2		
AK8	Regulation of cell energy metabolism	[37]
CCL2	Chemoattractant activity for monocytes, T cells, mast cells, and basophils	[51]
IFI44	Feedback regulation of host antiviral responses	[52]
IFI44L	Feedback regulation of host antiviral responses	[52]
**Under-expressed**		
CP	Related to different biological processes and diseases	[43]
SULF2	Related to different biological processes and diseases	[44]
ADSS1	Purine nucleotide synthesis	[49]
H2BC14	Nucleosome remodeling	[47]
MS4A6A	Related to different biological processes and diseases	[42]
SLC2A14	Proteins involved in carbohydrate transport	[53]
SLC2A3	Proteins involved in carbohydrate transport	[53]
SLC2A5	Proteins involved in carbohydrate transport	[53]
JCHAIN	Involved in the production of antibodies, playing a fundamental role in the binding of monomers	[54]
KLRB1	Regulation of immune responses and has been implicated in several diseases	[55]

**Table 3:** Comparing LbLRV1+ vs. LbLRV1-, among the top 10 overexpressed genes and the top 10 under-expressed genes.

Genes	Mechanism	Reference
**Overexpressed**		
IFIT1	Are related to the viral immune response and are addressed in the discussion	
IFIT2		
IFIT3		
ISG15		
IFITM3		
IFI44	Feedback regulation of host antiviral responses	[52]
IFI44L	Feedback regulation of host antiviral responses	[52]
MX1	Antiviral response of the human immune system	[56]
MX2	Antiviral response of the human immune system	[56]
USP18	Antiviral response of the human immune system	[57]
**Under-expressed**		
CAMP	Body’s defense against infections	[58]
SCIN	Body’s defense against infections	[58]
ANK3	Regulating the structure and stability of muscle cells	[59]
FBXO32	Regulating the structure and stability of muscle cells	[59]
HEY1	Regulation of the apoptosis process	[60]
TNFRSF10D	Regulation of the apoptosis process	[60]
AFF3	Transcription factors that are involved in the gene regulation process	[61]
JUN	Transcription factors that are involved in the gene regulation process	[61]
HS3ST2	Regulation of cellular processes such as cell adhesion, migration, and cell differentiation	[62]
PER3	Regulates the circadian cycle, metabolism, and other bodily function	[63]

The sets of genes with upregulation in the three groups of comparisons performed were subjected to enrichment analysis to evidence the active functional signaling pathways with significant value (adjusted P value < 0.05). In all, six libraries were used for initial enrichment (S4 Table). Of these, the most representative data banks were the *Go Biological Process 2022*, *WikiPathway 2021*, *BioPlanet 2019*, and *KEGG 2021 Human*.

From the gene lists obtained from the differential gene expression comparisons and the pathway enrichment analysis, the comparison LbLRV1+ vs. LbLRV1- was selected to continue the analysis. The other data generated were not addressed in this study.

**Fig 5:**
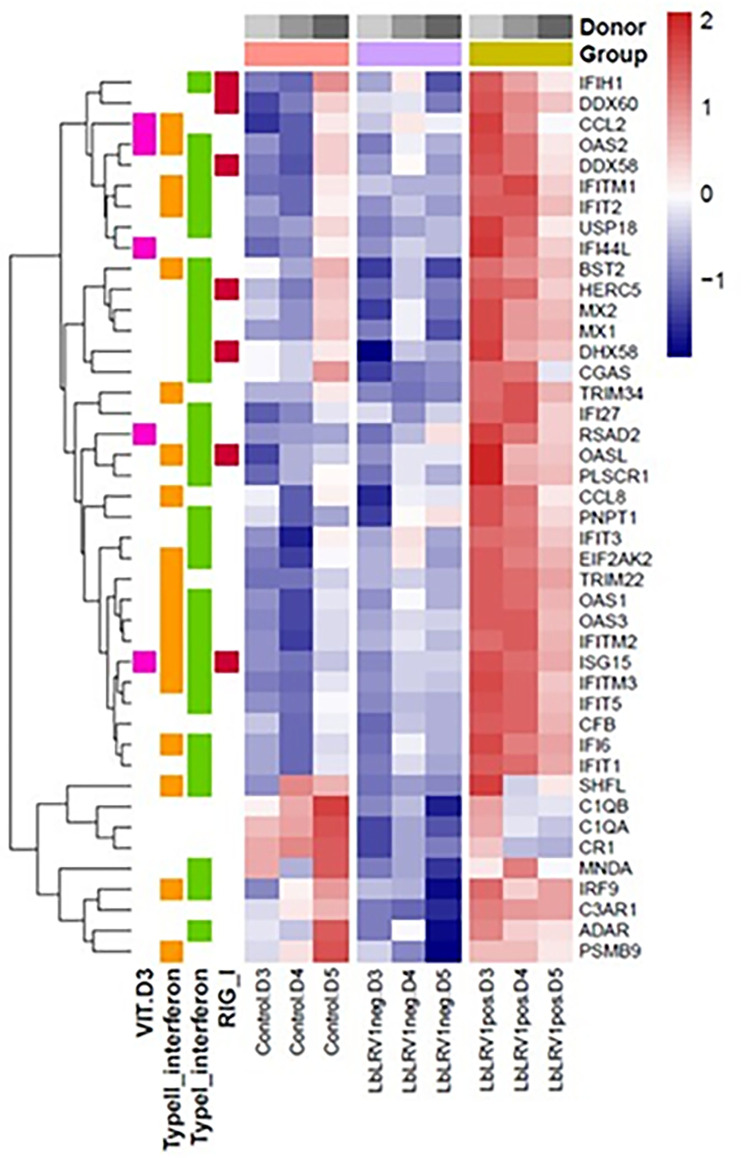
Heatmap of gene signature related to LRV1 presence. The row z-score values of normalized expression were generated to emphasize the difference of each gene between the samples/groups. The groups and donors are represented on the top-horizontal side, while the pathways related to each gene are represented on the left-vertical side. Hierarchical clustering was performed using the Euclidean distance to obtain gene clusters.

As can be seen in Fig 5, the heat map from the donors (D3, D4, and D5) showed overexpression of different genes related to the signaling pathways mediated by Interferons type I and II, by the RIG-I receptor, and by the non-genomic actions of vitamin D3, with differential expression in LbLRV1+ compared to the strain LbLRV1- and the control. It is worth noting that the differences observed in the microarray profiles between human macrophages infected with IOCL3626 (LbLRV1-) and IOCL3621 (LbLRV1+) may be influenced also by other differences observed between these two strains. Although we do not explore differences between these two *Leishmania* strains, they were both isolated from humans presenting CL, from the same geographic region and with a short time between the isolation of one strain and another, using the same in vitro conditions.

### 3.4. Gene expression by RT-qPCR of the selected signaling pathways from the transcriptome analysis of human monocyte-derived macrophages infected with LbLRV1+ or LbLRV1-

**Fig 6:**
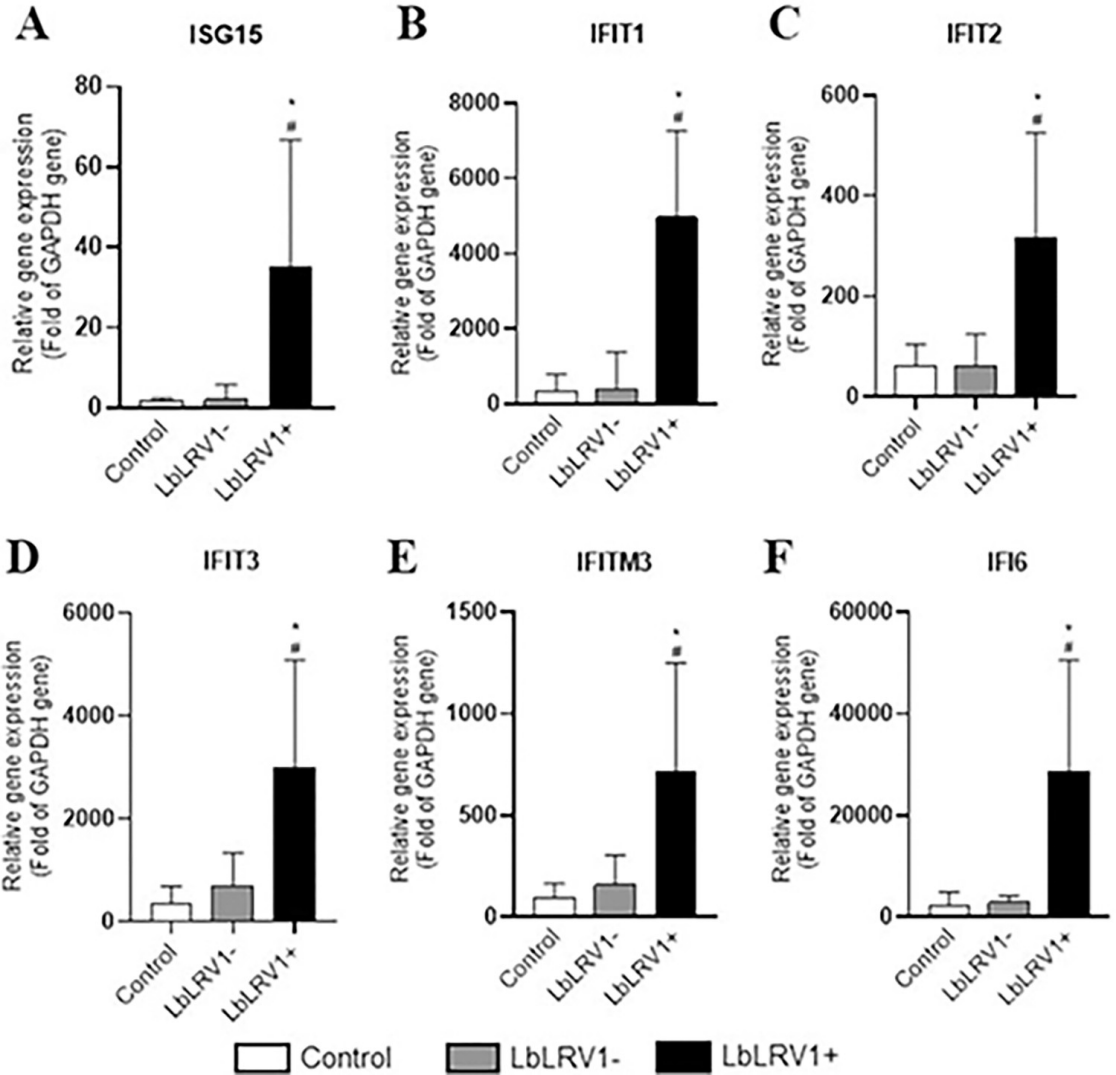
Relative analysis of mRNA expression of the genes from the IFNI signaling pathway using RT-qPCR. The ISG15, IFIT1, IFT2, IFT3, IFTM3, and IFI6 genes that were upregulated in the microarray were validated by RT-qPCR. The results were expressed as relative gene expression (Fold of GAPDH gene) and represent the mean ± SEM of three to five independent volunteers. *p<0.05 compared to control, and #p<0.05 compared to LbLRV1- (Data were presented with ANOVA followed by Tukey post-test).

From the INFI signaling pathway, the ISG15, IFIT1, IFT2, IFT3, IFTM3, and IFI6 genes that were upregulated in the microarray were validated by RT-qPCR. Results showed that all the studied genes were validated, and they were significantly highly expressed in human monocyte-derived macrophages infected with LbLRV1+ compared to LbLRV1- and control (Fig 6).

**Fig 7:**
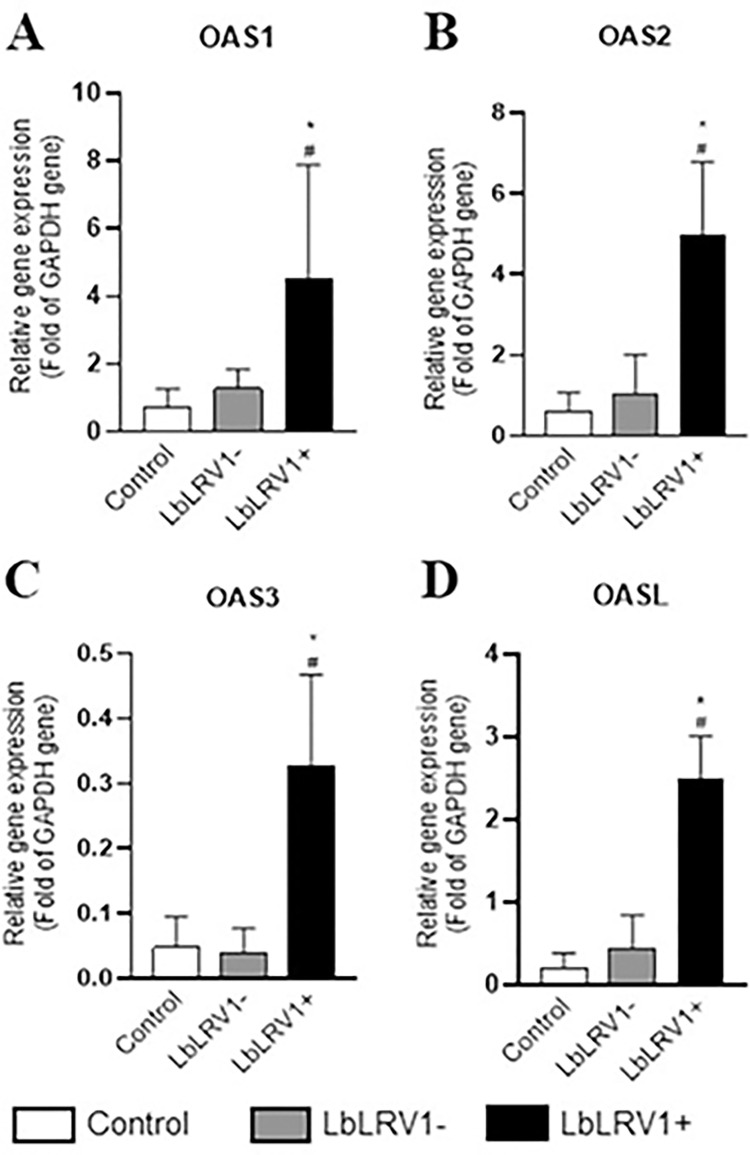
Relative analysis of mRNA expression of the OAS/RNase L oligoadenylate synthase signaling pathway using RT-qPCR. The OAS1, OAS2, OAS3, and OASL genes that were upregulated in the microarray were validated by RT-qPCR. The results were expressed as relative gene expression (Fold of GAPDH gene) and represent the mean ± SEM of three to five independent volunteers. *p<0.05 compared to control, and #p<0.05 compared to LbLRV1- (Data were presented with ANOVA followed by Tukey post-test).

From the OAS/RNase L oligoadenylate synthase signaling pathway, the OAS1, OAS2, OAS3, and OASL genes that were upregulated in the microarray were also validated by RT-qPCR. Results showed that all the studied genes were validated, and they were significantly highly expressed in human monocyte-derived macrophages infected with LbLRV1+ compared to LbLRV1- and control (Fig 7).

### 3.5. Cytokines quantification from the supernatant of the human monocyte-derived macrophages infected with LbLRV1+ or LbLRV1-

**Fig 8:**
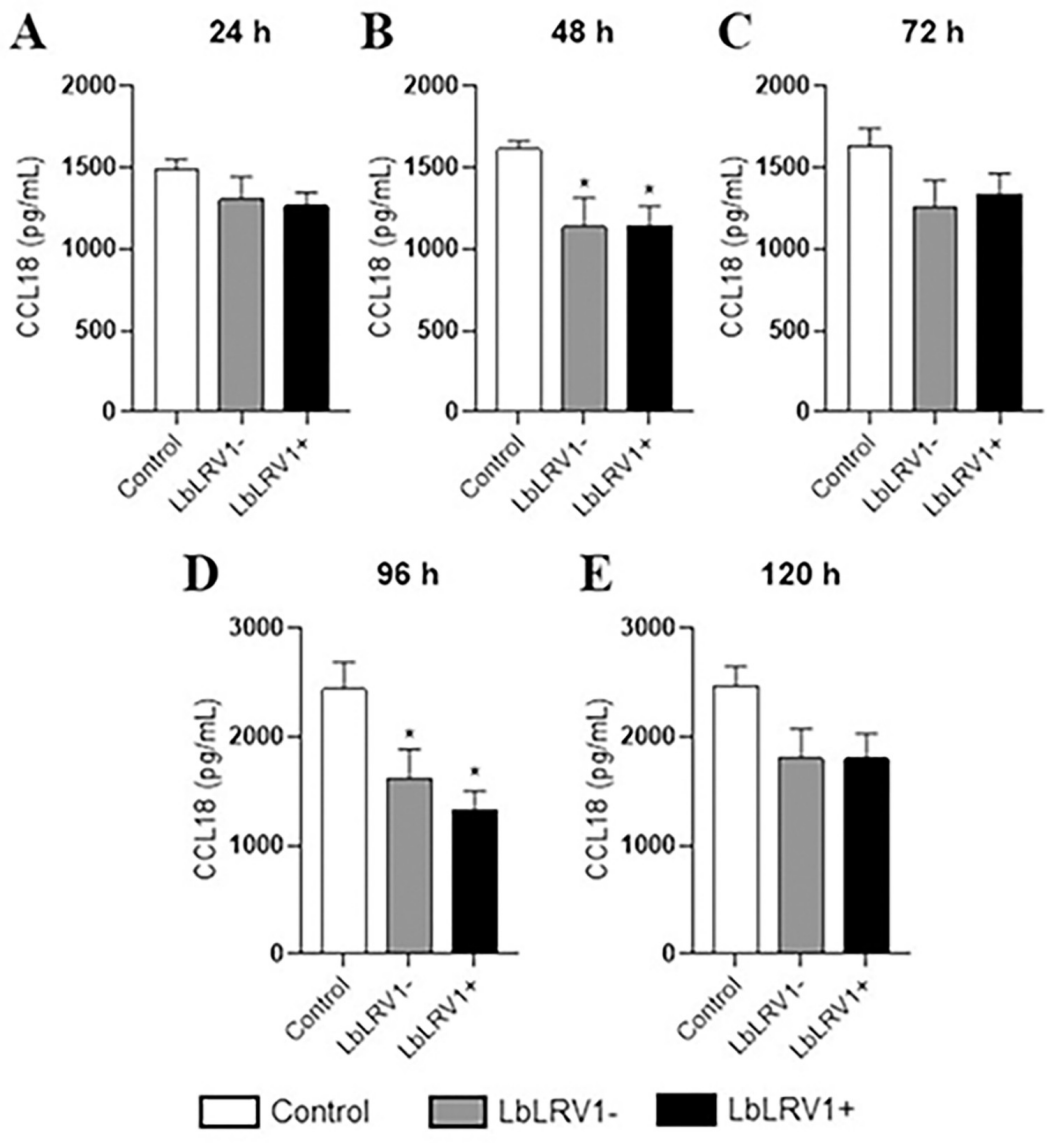
CCL18 quantification by ELISA. Supernatant from the incubation of human monocyte-derived macrophages infected with LbLRV1+, LbLRV1- or without *Leishmania* (control) was used to determine the cytokines levels. The results were expressed as pg/mL of CCL18 produced and represent the mean ± SEM of three to five independent volunteers. *p<0.05 compared to control, and #p<0.05 compared to LbLRV1- (Data were presented with ANOVA followed by Tukey post-test).

The supernatant of the infection of human monocyte-derived macrophages infected with LbLRV1+, LbLRV1- or without *Leishmania* was used to determine the cytokines levels. It was observed a decrease in CCL18 levels in the infected cells compared to the control after 48 and 96 h of infection (Fig 8). No significant differences were found in IFN-α and IFN-β cytokines levels between LbLRV1+ vs. LbLRV1- and the control in the assays.

**Fig 9:**
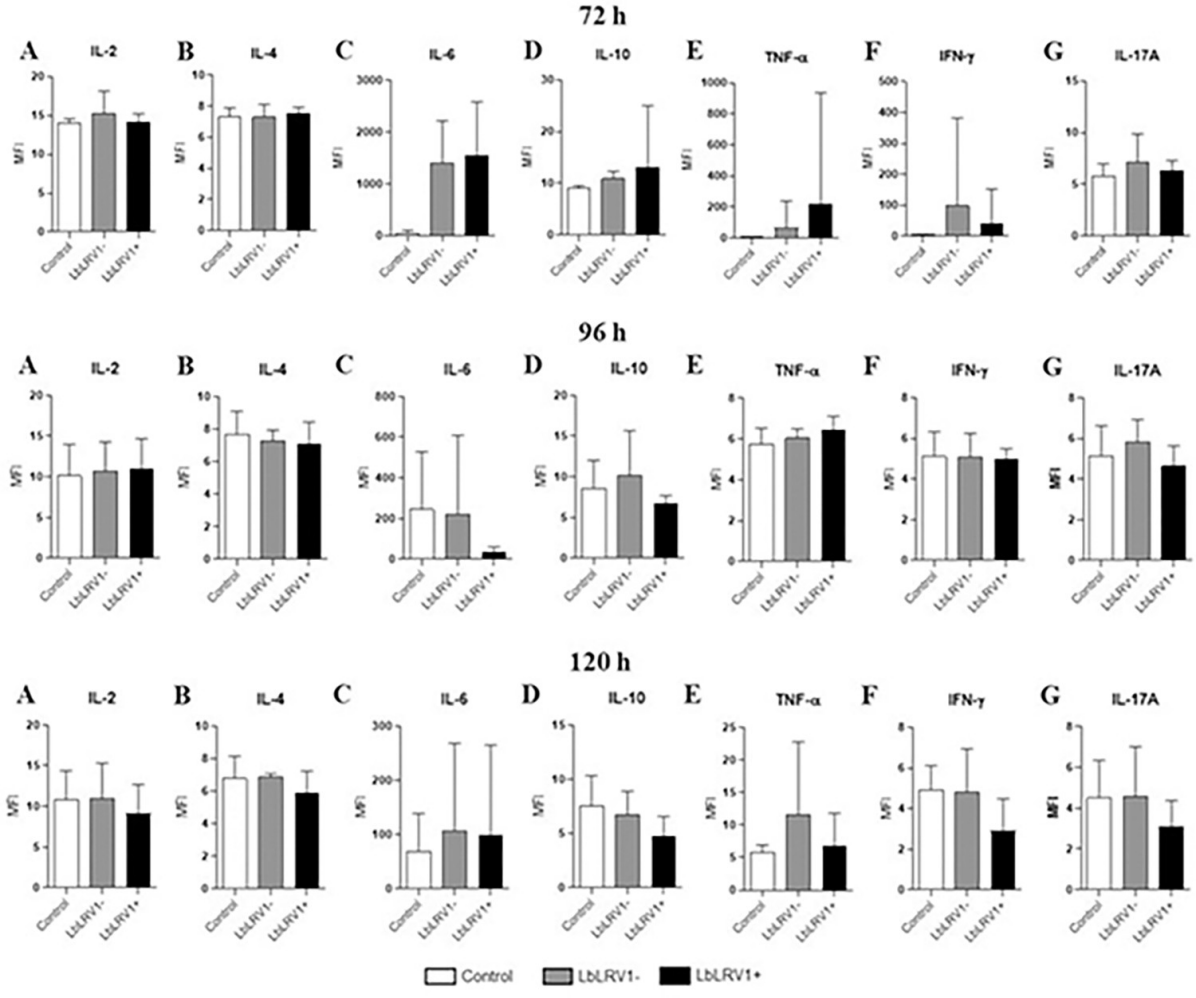
Th1, Th2, and Th17 cytokines quantification by CBA. Supernatant from the incubation of human monocyte-derived macrophages infected with LbLRV1+, LbLRV1- or without *Leishmania* (control) was used to determine the cytokines levels. The results were expressed as mean fluorescence intensity (MFI) and represent the mean ± SEM of three to five independent volunteers. *p<0.05 compared to control, and #p<0.05 compared to LbLRV1- (Data were presented with ANOVA followed by Tukey post-test).

The cytokines from Th1, Th2, and Th17 phenotypes were also evaluated. However, no significant differences were found in cytokines levels between LbLRV1+ vs. LbLRV1- and the control in the CBA assay at different periods evaluated (Fig 9).

**Fig 10:**
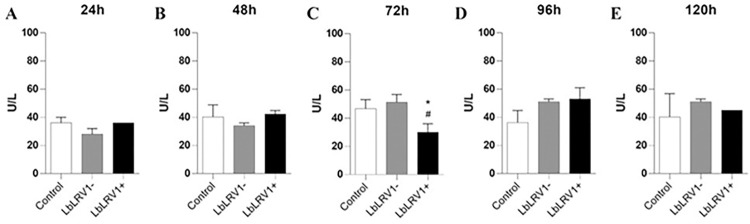
LDH quantification. Supernatant from the incubation of human monocyte-derived macrophages infected with LbLRV1+, LbLRV1- or without *Leishmania* (control) was used to determine the LDH levels. The results were expressed as U/L of LDH produced and represent the mean ± SEM of three to five independent volunteers. *p<0.05 compared to control, and #p<0.05 compared to LbLRV1- (Data were presented with ANOVA followed by Tukey post-test).

The LDH levels were also evaluated in the supernatant of the incubation of human monocyte-derived macrophages infected with LbLRV1+, LbLRV1- or without *Leishmania* (control). In Fig 10, there was no significant difference between the samples, except for the evaluation point of 72 h of incubation. This suggests that, in most cases, the cells remained viable throughout the period evaluated, with the only exception occurring after 72 h of incubation.

## 4. Discussion

The findings of the initial exploratory analyses with the microarray results provide evidence that supports the contribution of LRV1 for controlling the host immune response following infection. Even with published research highlighting the significance of LRV1 detection in TL cases caused by *L*. *braziliensis*, the signaling pathways that were triggered remain mostly unknown. The analysis reveals various genes that are overexpressed in cells infected with the LbLRV1+ strain compared to cells infected without the LbLRV1- strain and the control. These genes are associated with signaling pathways controlled by type I and II interferons.

The data suggest the activation of signaling pathways associated with the presence of LRV1 and include genes from pathways not yet reported so far. This study shows for the first time the activation of the OAS/RNase L signaling pathway and the non-genomic actions of vitamin D3 when comparing the human monocyte-derived macrophages infected with LbLRV1+ vs. LbLRV1- and the control. Initial results reinforce the role of LRV1 in directing the host’s immune response after infection and its identification in patients with TL may be useful in screening to assess disease progression.

Herein, the incubation of human monocyte-derived macrophages with LbLRV1+ or LbLRV1- demonstrated a higher percentage of infection and phagocytic index in cells infected with the positive strain, the LRV1, after 72 h. This data reinforces previous studies that report the influence of LRV1 in increasing the replication and survival rate of *L*. (*V*.) *guyanensis* in experimental models [64–66]. LRV1 seems to favor both the infection and the survival of the parasite inside macrophages. However, the factors involved in this process are still unknown [67,68]. A valid point to be considered regarding the percentage of infection, phagocytic index in infected cells and differences observed in the microarray profiles between human macrophages infected with IOCL3626 (LbLRV1-) and IOCL3621 (LbLRV1+) is that the cultures are not isogenic, that is, there may be a potential influence of the different genotypes of the parasites [25].

Given the results presented here, other studies can be conducted with other parasites containing LRV1, even from other species, to verify whether the same pathways are important in infections caused by other etiological agents that also carry LRV1, which, as we know, vary in function of the *Leishmania* species that they infect [69]. Another study alternative would be to eliminate LRV1 and thus compare the same strain with LRV1 and cure since the LRV1 elimination treatment does not seem to affect the biology of parasites of the Viannia subgenus [70]. However, the present study presents a series of results that direct further studies.

In general, *L*. *braziliensis* infections are characterized by an inflammatory response mediated by CD4+ T cells that produce IFN-γ, an interferon type II, responsible for the activation of macrophages and subsequent death of intracellular parasites. [15] showed the differential expression of immune response genes associated with IFN-γ production in *L*. *braziliensis* infection, and these genes were related to the innate immune response, such as the CXCL10 and IL1B genes which were also observed in microarray results with the same species corroborating with the present data. However, despite the two analyses addressing parasites of the same species, it is important to emphasize that in the region of the study of [15], the viral endosymbiont inside the parasites was not observed, in addition to the genotypic differences in the parasites of the *L*. *braziliensis* species that circulate in the two regions [71–73]. The presence of LRV1 has been associated with the development of more severe forms of leishmaniasis, including relapses after treatment [16,74–76].

The relationship between the virus and the development of severe forms is dependent on the production of Type I Interferon (Type I IFN) by macrophages, since, by infecting mice with *L*. *guyanensis* parasites they are cured of LRV1 and then performing a treatment with type I IFN, an increase in lesion size and parasite load was observed, reproducing the phenotype of infection with *L*. *guyanensis* LRV1+. The model was further tested with other exogenous viral infections that induce type I IFN response, confirming previous findings and reinforcing that parasites co-infected with LRV1 provide a significant risk factor. In the present work, the analysis of the incubation of human monocyte-derived macrophages infected with LbLRV1+, LbLRV1- or without *Leishmania* (control), a significant increase in the expression of different genes of type I and II Interferon signaling pathways was observed when compared with the data from the LbLRV1+ samples vs. LbLRV1- and the control. The data corroborate the aforementioned information and emphasize the importance of this signaling pathway in the development of the disease and its possible relationship with LRV1.

The expression of genes ISG15, IFIT1, IFIT2, IFIT3, IFITM3, and IFI6 of the IFNI signaling pathway and OAS1, OAS2, OAS3, and OASL of the oligoadenylate synthase signaling pathway showed significant overexpression by RT-qPCR when comparing LbLRV1+ vs. LbLRV1- and the control at 72 h, which corroborates the results found in the microarray assay.

No significant differences were found in IFN-α and IFN-β cytokine levels between LbLRV1+ vs. LbLRV1- and the control in the ELISA assay at 24 up to 120 h. In the ELISA quantification for CCL18, significant differences were identified in the comparisons between the infected and control at 48 and 96 h after infection. In leishmaniasis, the CCL18 is related to the polarization of M2 macrophages, negative regulation of pro-inflammatory cytokines, and recruitment of Th2 cells [77]. Cytokines of Th1, Th2, and Th17 phenotypes were also evaluated. However, no significant differences were found in cytokine levels between LbLRV1+ vs. LbLRV1- and the control in the CBA assay at 24 up to 120 h. The results corroborate with the data published in the literature regarding the frequent M1/M2 dichotomy in individuals affected with leishmaniasis [78].

The supernatant from the incubation of human monocyte-derived macrophages infected with LbLRV1+, LbLRV1- or without *Leishmania* was also used to measure LDH levels in response to infection to determine cell viability. Pyroptosis is recognized as a pro-inflammatory type of cellular death that is mediated by Gasdermin D (GSDMD), a protein that is dependent on CASPASE-1 and is consequently connected to the activation of the NLRP3 inflammasome complex [79]. At 24 up to 120 h, no significant differences in LDH release were observed in all groups. At 72 h, cells infected with LbLRV1+ showed a decrease in LDH release compared to infection with LbLRV1- and control. It is important to note that control PBMCs liberate around 40 U/L of LDH which is according to literature [80]. In addition, [81] evaluated the LDH release at 2 and 24 h after infection and found no significant differences.

In a study carried out with *L*. *amazonensis* in murine macrophages, it was demonstrated that Gasdermin D is activated in the initial stages of infection (2 h), which leads to transient cell permeabilization and potassium efflux, promoting the activation of the NLRP3 inflammasome. At 24 h, the level of Gasdermin D was higher in uninfected cells compared to infected ones [81]. In the present study, when comparing LbLRV1+ vs. control and LbLRV1- vs. control, significant negative regulation for the Gasdermina E gene was identified at 72 h, independent of the presence of LRV1. These data support the idea of transient membrane permeabilization in the early post-infection period.

### Non-genomic actions Of 1.25 dihydroxy VITAMIN D3

Vitamin D3 (25D) is essential for maintaining calcium homeostasis. In recent years, literature has demonstrated its role in controlling the function of the human immune system by providing protection against pathogens and preventing the harmful effects that can occur due to a prolonged inflammatory response [82,83]. 25D signals through a specific nuclear receptor (VDR) and its functions are characterized as genomic and non-genomic.

1-α-hydrolase (CYP27B1) is part of the cytochrome P450 superfamily of enzymes and is responsible for catalyzing 25D to the active form 1.25-dihydroxy vitamin D3/calcitriol (1,25D) to act autocrine or paracrine way when detection of microbial infection by macrophages occurs [84]. Thus, 1,25D binds to the VDR and acts as a regulator of gene transcription with co-activator or co-repressor action, depending on the target gene [85].

The present study is in line with those described in the literature since there is the activation of the signaling pathway of the non-genomic actions of 1.25D due to an increase in CD40, TLR-2, and TLR-4 gene expression in LbLRV1+ when compared to LbLRV1-. These genes are involved in stimulating CYP27B1 production and 25D activation. These results are consistent with previous studies that demonstrate a correlation between the expression level of TLR-2/4 and the mitochondrial enzyme CYP27B1 [86–88] and the CD40 ligand and CYP27B1 in human monocytes [89].

The active metabolite 1.25D modulates the growth, differentiation, and function of different cells of the immune system [84]. In macrophages, the production of 1.25D is constantly deregulated, which can culminate in the accumulation of the metabolite and favor the appearance of hypercalcemic complications [90]. Inadequate levels of 25D have been correlated with increased susceptibility to autoimmune diseases and infections [84]. This evidence provides support for the study by [87], which proposed that 25D increases the ability of macrophages to eliminate *Mycobacterium tuberculosis* through the upregulated expression of antimicrobial peptides.

On the other hand, monocytes isolated from patients with dermal sequelae of visceral leishmaniasis (post-kala-azar dermal leishmaniasis—PKDL) demonstrated decreased expression of TLR-2/4, simultaneous attenuation of ROS and increased expression of classic M2 markers (CD206, ARG1, and PPARγ). In addition, the presence of alterations in the 25D signaling pathway was considered a key feature in the presentation of PKDL, since the development of the disease was correlated with elevated plasma levels of 25D and associated genes, which suggests a relationship between M2 polarization and the 25D signaling pathway [91]. It is important to emphasize that the clinical presentation of PKDL is associated with *Leishmania donovani* and is very different from TL caused by *L*. *braziliensis*.

In addition to the activation of the 1.25D non-genomic actions signaling pathway described, this study suggests that the expression of the antiviral genes IFI44L, ISG15, OAS1, OAS2, OAS3, and RSAD2 occurs via the INF-α–JAK1 –STAT1/2 triggered in the presence of LRV1.

Taken together, the results of the present study point to a probable modulation of the 25D-dependent immune response in human infections caused by *L*. *braziliensis* co-parasitized by LRV1, since macrophage infections with LbLRV1- did not lead to gene expression of CD40, TLR-2, and CP27B1.

### Oligoadenylate synthase OAS/RNASE L signaling pathway

The innate immune system is part of a critical and highly complex process aimed at limiting the infection of cells. After a successful viral infection, for example, the cell is signaled to enter its antiviral state, starting to present high resistance to these microorganisms, rapidly favoring the apoptotic process in response to a new viral infection [92].

When a cell’s viral state is activated, the transcription levels of about 300 genes are increased together. Among the increased enzymes is Ribonuclease L (RNASEL), which despite being present in small amounts during the normal cell cycle, is found in its inactive form [93]. Together they increase the transcription of a set of genes encoding 2’-5’ OAS. The transcribed OAS RNA undergoes important post-transcriptional and post-translational changes that will determine its location in the cell and its length [94,95].

The 2’-5’ OAS/RNAse L system favors host protection against pathogenic viruses by cleaving dsRNA into small 2’-5’A oligonucleotides, an early transient response molecule that acts as a messenger for RNASEL activation [96]. Herein, the present study provides support regarding the role of the OAS antiviral response in the presence of LRV1+ compared to LRV1- in *L*. (*V*.) *braziliensis*, as the genes OAS1, OAS2, OAS3, OASL, and DDX58 are overexpressed.

On the other hand, ABCE1 and RNASEL genes are negatively regulated, which suggests modulation of the signaling pathway to subvert the mechanism that limits viral replication, developing alternative antiviral functions of RNASEL-independent OAS proteins, as seen by [97]. A study was carried out in mice, which demonstrated the association of an OAS1b allele with the phenotype of resistance to infection by the Yellow Fever virus, belonging to the genus Flavivirus and causing yellow fever [98].

Additionally, the presented data are consistent with another study, which suggests the existence of at least two independent RNASEL pathways through which OAS genes can develop antiviral activity [99]. Studies performed in mice provide evidence of two mechanisms to suppress flavivirus replication: through the classical pathway mediated by RNA cleavage by RNase L [100] and through the non-classical mechanism that is not well defined, but is mediated by the gene of FLV resistance, resistant allele of the OAS1b protein [98].

Previous studies suggest the action of ABCE1 together with RNAse L to eliminate exogenous RNA [96]. Taken together, the present data provide evidence for the regulation of the 2’-5’ OAS pathway independent of ABCE1 and RNAse L, providing support for a key role of this pathway in the immune response in infections by LbLRV1+ parasites.

### Retinoic acid-inducible gene I-like receptor signaling pathway

Retinoic acid-inducible gene I-like receptors (RLRs) are a family of cytosolic helicase enzymes that act as sensors in the detection of double-stranded RNA and modulation of cellular immunity. After activation, these sensors lead to the induction of transcription mainly of genes that encode IFNI and antiviral genes and inflammasome activation [101]. In terms of signaling, RLRs are like TLRs because they detect viral RNA and induce ISG, IFNI, and pro-inflammatory cytokines. However, TLRs detect nucleic acids that have entered the cell via endocytosis while RIG-I and MDA5-type RLRs detect intracellular viral RNA in active replication [102].

Although these receptors are primarily known for their role in the immune response to RNA viruses, recent studies suggest that they may be involved in the immune response to other infections, including parasitic and DNA virus infections [103,104]. In this study, the data presented are consistent with the literature, since in the comparison of LbLRV1+ vs. LbLRV1, the IFIH1, DDX58, DHX58, and ISG15 genes that are part of the RIG-I type RLR receptor signaling pathway are overexpressed.

On the other hand, [105] investigated the role of inflammasome-dependent and inflammasome-independent cytoplasmic dsRNA sensors, RLRs, and NLRs in a murine model of *L*. *guyanensis* infection and did not find any role for these inflammasome-dependent sensors, regardless of the presence of LRV1.

Together, the data described here, and another recent study [106] suggest that RIG-I-type receptors may be involved in the immune response to *L*. *braziliensis* infection, however, additional research is needed to demonstrate parasite recognition and whether its activation is secondary to other stimuli, such as IFNI production in response to infection or the presence of the viral endosymbiont LRV1.

## 5. Conclusion

Herein the proposed experimental model was standardized and proved to be satisfactory, providing enough material to carry out the microarray methodology from the separation of cells from human donors submitted to infection with *Leishmania braziliensis* with and without LRV1. In general, and still exploratory, it was possible to observe the participation of several genes already described in the literature in important processes for the development of the disease, which in a way makes valid the used approach.

Specifically on the alternative hypothesis, differentially expressed genes were observed in comparative analyses with LbLRV1+ infections. Limitations of the study include the small number of blood donors (n = 3) used in gene expression profiles, the potential influence of different parasite genotypes, since the strains are not isogenic, with only one positive and one negative strain for LRV1 used and a limited number of genes were selected for validation by other methodologies. Despite these important limitations, the results reinforce the role of LRV1 in the host immune response in the early stages of infection, with differential expression of several genes related to Type I and Type II Interferon signaling pathways, non-genomic actions of 1.25 dihydroxy vitamin D3, OAS /RNAse L, and RIG-like receptors.

Other differentially expressed genes (positively and negatively) that were not addressed here will be made available later in a descriptive way, which may help in the development and/or direction of future studies.

## Supporting information

S1 Fig Analysis of the population of mononuclear cells obtained from human peripheral blood. (A) Dot plot size (FSC) versus granularity (SSC) of cell viability, (B) viable and non-viable cells using TO/PI Staining, (C) Dot plot size (FSC) versus granularity (SSC) for monocyte population analysis, and (D) whole blood gated PBMC population was labeled with anti-CD14-PE.
(TIFF)

S1 Table All differentially expressed genes are analyzed by each comparison and unique genes are represented in the Venn diagram. (XLSX)

S2 Table Results regarding pathway enrichment for each comparison. (XLSX)

S3 Table Primer sequences for gene-specific amplification. F: Forward Primer; R: Reverse Primer.
(DOCX)

S4 Table Libraries used for the enrichment of the metabolic pathways. (DOCX)
