# Supplementary figures and images for: Transcriptomics analysis highlights potential ways in human pathogenesis in Leishmania braziliensis infected with the viral endosymbiont LRV1
Source: PLoS Negl Trop Dis. 2024 May 14;18(5):e0012126. doi: 10.1371/journal.pntd.0012126 (PMC11093365; doi:10.1371/journal.pntd.0012126)

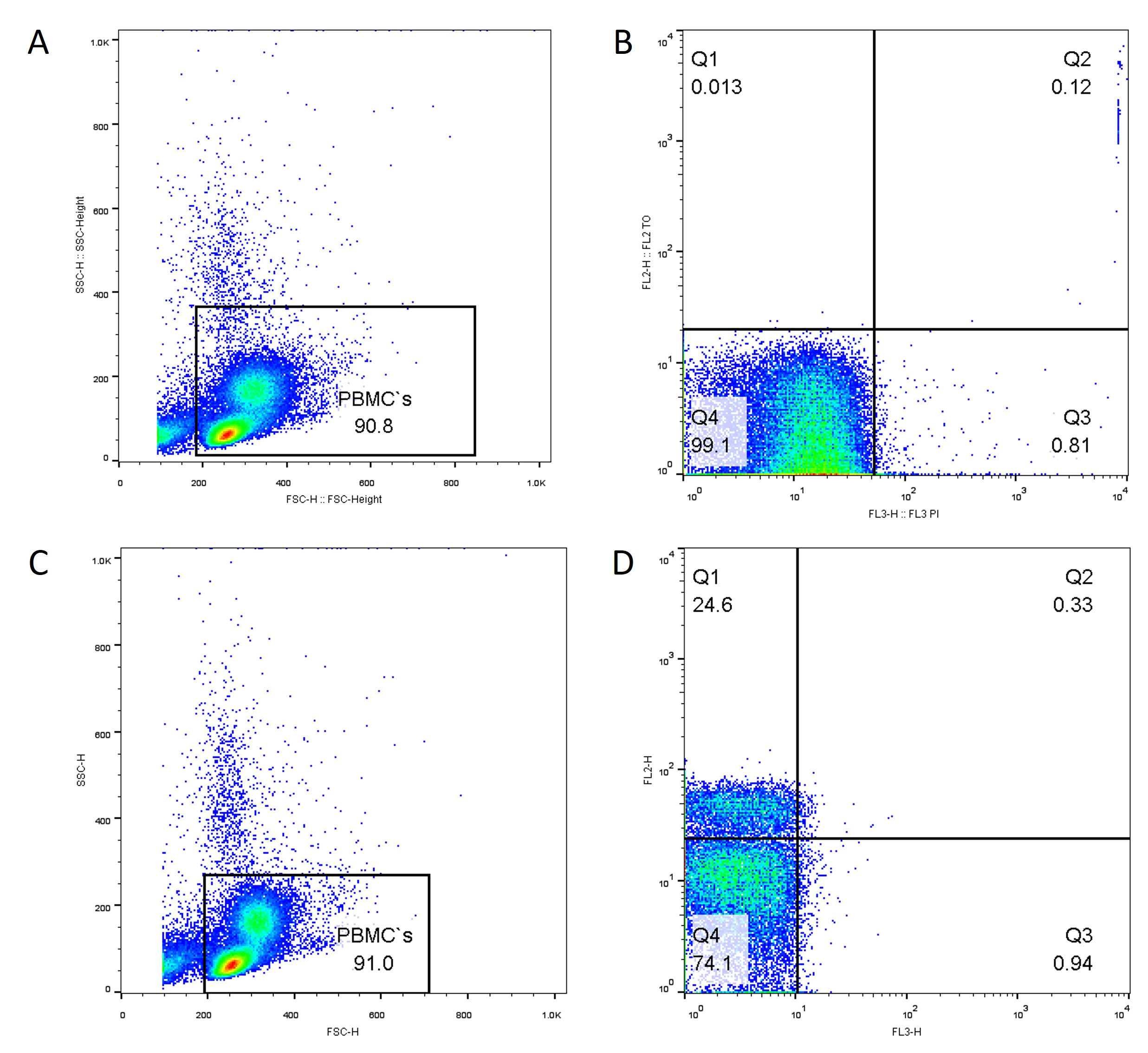

Supplement: S1 Fig — (A) Dot plot size (FSC) versus granularity (SSC) of cell viability, (B) viable and non-viable cells using TO/PI Staining, (C) Dot plot size (FSC) versus granularity (SSC) for monocyte population analysis, and (D) whole blood gated PBMC population was labeled with anti-CD14-PE. (TIFF) [file pntd.0012126.s001.tiff]
