# Supplementary material for: Transcriptomics analysis highlights potential ways in human pathogenesis in Leishmania braziliensis infected with the viral endosymbiont LRV1
Source: PLoS Negl Trop Dis. 2024 May 14;18(5):e0012126. doi: 10.1371/journal.pntd.0012126 (PMC11093365; doi:10.1371/journal.pntd.0012126)
Supplement: S3 Table — F: Forward Primer; R: Reverse Primer. (DOCX) [file pntd.0012126.s004.docx]

**Table S3 - Primer sequences for gene-specific amplification.**

| Gene ID | Description | Sequence | Efficiency (%) | PrimerBank ID |
| --- | --- | --- | --- | --- |
| ISG15 | Homo sapiens ISG15 ubiquitin-like modifier | F: TGGACAAATGCGACGAACCTC  R: TCAGCCGTACCTCGTAGGTG | 99 | 193083170c2 |
| IFIT1 | Homo sapiens interferon-induced protein with tetratricopeptide repeats 1 | F: GCGCTGGGTATGCGATCTC  R: CAGCCTGCCTTAGGGGAAG | 99 | 116534936c2 |
| IFIT2 | Homo sapiens interferon-induced protein with tetratricopeptide repeats 2 | F: GACACGGTTAAAGTGTGGAGG  R: TCCAGACGGTAGCTTGCTATT | 99 | 153082754c3 |
| IFIT3 | Homo sapiens interferon-induced protein with tetratricopeptide repeats 3 | F: AGAAAAGGTGACCTAGACAAAGC  R: CCTTGTAGCAGCACCCAATCT | 101 | 197276657c3 |
| IFITM3 | Homo sapiens interferon induced transmembrane protein 3 | F: TGCCTGGGCTTCATAGCATT  R: TCACAGGGACACACAAGTCC | 104 | 10835238a1 |
| IFI6 | Homo sapiens interferon, alpha-inducible protein 6, transcript variant 3 | F: GGTCTGCGATCCTGAATGGG  R: TCACTATCGAGATACTTGTGGGT | 98 | 94538330c1 |
| OAS1 | Homo sapiens 2'-5'-oligoadenylate synthetase 1, 40/46kDa | F: AGCTTCGTACTGAGTTCGCTC  R: CCAGTCAACTGACCCAGGG | 104 | 74229012c2 |
| OAS2 | Homo sapiens 2'-5'-oligoadenylate synthetase 2, 69/71kDa | F: CTCAGAAGCTGGGTTGGTTTAT  R: ACCATCTCGTCGATCAGTGTC | 104 | 74229020c1 |
| OAS3 | Homo sapiens 2'-5'-oligoadenylate synthetase 3, 100kDa | F: GAAGGAGTTCGTAGAGAAGGCG  R: CCCTTGACAGTTTTCAGCACC | 104 | 45007006c1 |
| OASL | Homo sapiens 2'-5'-oligoadenylate synthetase-like, transcript variant 1 | F: CTGATGCAGGAACTGTATAGCAC  R: CACAGCGTCTAGCACCTCTT | 98 | 38016933c1 |
| GAPDH | Homo sapiens glyceraldehyde-3-phosphate dehydrogenase (GAPDH), transcript variant 2 | F: GGAGCGAGATCCCTCCAAAAT  R: GGCTGTTGTCATACTTCTCATGG | 99 | 378404907c1 |

F: Forward Primer; R: Reverse Primer.
