# Supplementary material for: Transcriptomics analysis highlights potential ways in human pathogenesis in Leishmania braziliensis infected with the viral endosymbiont LRV1
Source: PLoS Negl Trop Dis. 2024 May 14;18(5):e0012126. doi: 10.1371/journal.pntd.0012126 (PMC11093365; doi:10.1371/journal.pntd.0012126)
Supplement: S4 Table — (DOCX) [file pntd.0012126.s005.docx]

**Table S4 – Libraries used for the enrichment of the metabolic pathways.**

|  | **LbLRV1- vs. control** | | **LbLRV1+ vs. control** | | **LbLRV1+ vs. LbLRV1-** | |
| --- | --- | --- | --- | --- | --- | --- |
| **LIBRARY** | ***UP*** | ***DOWN*** | ***UP*** | ***DOWN*** | ***UP*** | ***DOWN*** |
| *Go Biological Process 2022* | 02 | 117 | 121 | 106 | 127 | 0 |
| *WikiPathway 2021* | 0 | 34 | 11 | 35 | 16 | 0 |
| *BioPlanet 2019* | 0 | 79 | 43 | 148 | 18 | 0 |
| *KEGG 2021 Human* | 02 | 48 | 09 | 71 | 23 | 0 |
| Reactome 2016 | 0 | 62 | 138 | 48 | 12 | 0 |
| MSigDB Halmark 2020 | 9 | 35 | 11 | 14 | 21 | 03 |
